# Body fluids biomarkers associated with prognosis of acute ischemic stroke: progress and prospects

**DOI:** 10.2144/fsoa-2023-0142

**Published:** 2024-05-20

**Authors:** Fengmang Jiang, Junhua Li, Simin Yu, Jinli Miao, Wenmin Wang, Xiaohong Xi

**Affiliations:** 1 Emergency Intensive Care Unit, The Quzhou Affiliated Hospital of Wenzhou Medical University, Quzhou People's Hospital, Quzhou, 324000, PR China; 2 Biological Medicine Research & Development Center, Yangtze Delta of Zhejiang, Hangzhou, 314006, PR China

**Keywords:** acute ischemic stroke, biomarker, body fluid, prognosis, review

## Abstract

Acute ischemic stroke (AIS) is one of the most common strokes posing a grave threat to human life and health. Predicting the prognosis of AIS allows for an understanding of disease progress, and a better quality of life by making individualized treatment scheme. In this paper, we conducted a systematic search on PubMed, focusing on the relevant literature in the last 5 years. Summarizing the candidate prognostic biomarkers of AIS in body fluids such as blood, urine, saliva and cerebrospinal fluid is often of great significance for the management of acute ischemic stroke, which has the potential to facilitate early diagnosis, treatment, prevention and long-term outcome improvement.

## Plain language summary

Acute ischemic stroke stands as a prominent contributor to global mortality and disability rates. This comprehensive review delves into the present state and advancements in the study of prognostic biomarkers for AIS in body fluids, enabling the monitoring of disease progression and prediction of prognosis. Furthermore, we elucidate the utilization of multiple biomarkers to predict outcomes more precisely. This paper emphasizes the importance of predicting disease progression as early as possible so that clinicians can change treatment regimens in time to better treat their patients.

## Abstract (plain language summary)

Acute ischemic stroke is a major public health problem facing the world acute ischemic stroke (AIS) is one of the leading causes of death and disability worldwide.

The prognosis of AIS is bleak due to the limited availability of effective treatments and the short time window for treatment.

Patient outcomes could be improved through prognostic markers for AIS.

There are various risk or protective factors that affect the prognosis of AIS. Early detection of prognostic markers in AIS patients is of great significance for the prognostic management of patients.

Moreover, some prognostic markers are also important therapeutic targets for AIS, and early regulation of prognostic markers can effectively improve the prognosis of AIS patients.

Research progress on body fluid prognostic markers in AIS.

There are many biomarkers in the blood, urine, saliva and cerebrospinal fluid of AIS patients that can effectively evaluate the patient's prognosis.

The prognostic markers in blood are mainly cell count, and the prognostic value of the most classic neutrophils-to-lymphocytes ratio has been widely confirmed.

In addition, great progress has been made in assessing the prognosis of AIS by detecting the expression of some inflammation-related cytokines and marker proteins as well as specific genes.

Due to the low cost of testing and easy access to samples, blood-derived prognostic markers are among the easiest to translate and apply. The prognostic value of urine protein and urine osmolality is also in the initial stage of exploration.

Interestingly, we found that some studies reported that microorganisms, inflammatory cytokines, purines, etc. in saliva are related to the prognosis of AIS patients and showed excellent prognostic performance.

Inflammatory cytokines, microtubule-associated protein tau, glial fibrillary astrocytic protein, etc. in cerebrospinal fluid are also closely related to the prognosis of AIS. Development direction of body fluid prognostic markers in AIS.

There is a need to further determine the consistency and superiority of AIS biomarkers from different body fluids.

In addition, mining effective therapeutic targets from prognostic markers is an urgent and extremely important research content.

Acute ischemic stroke (AIS) is a common type of stroke defined as a neurological disorder resulting from ischemic death of local brain, spinal cord or retinal cells (Figure 1A) [1]. AIS accounts for nearly 90% of all strokes and is one of the leading causes of death and disability. Approximately 800,000 people experience new or recurrent strokes each year. Among these cases, approximately 87% are ischemic, 10% intracranial hemorrhage and 3% subarachnoid hemorrhage [2]. Therefore, AIS remains a key global public health problem. From 1990 to 2019, there was a substantial rise in both the prevalence and fatality rate of stroke in China. The number of reported stroke cases surged from 1.76 million to 3.93 million, while the mortality rate due to stroke escalated from 1.38 million to 2.19 million [^3-5^]. In the meantime, the number of stroke deaths worldwide increased from 2.04 million to 3.29 million and that is expected to further increase to 4.9 million by 2030, which posing a considerable burden on both society and individuals [6]. Guidelines updated in 2018 by the American Heart Association/American Stroke Association (AHA/ASA) recommend using the National Institutes of Health Stroke Scale (NIHSS; minor, ≤5; mild, 6–10; moderate, 11–15; and severe, ≥16) to assess stroke severity [7]. NIHSS is a 15-item neurological checklist that assess changes in stroke severity and clinical status, with scores ranging from 0 to 42. Higher scores of NIHSS indicate higher stroke severity. Current AIS treatment strategies include restoring blood flow and a range of medical, endovascular and surgical strategies. When applied in a timely and appropriate manner, it can prevent secondary deterioration caused by brain and systemic complications, as well as recurrent stroke, and improve short-term and long-term outcomes. Since treatment of AIS is time-dependent, treatment executed in the early stage of AIS can reduce morbidity and mortality. Therefore, rapid detection and diagnosis of AIS patients is essential to improve their prognosis. Clinicians need to adjust intervention measures according to individual conditions, and then effectively improve the prognosis of patients.

**Figure 1.**
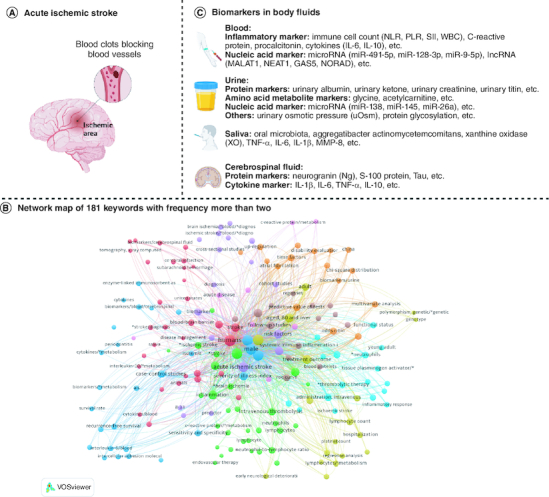
The candidate prognostic biomarkers of AIS in body fluids. **(A)** Schematic diagram of AIS. **(B)** Network map of 181 keywords, these 181 keywords that appeared more than twice were included and classified into nine clusters in the map by the VOS viewer. **(C)** AIS prognostic biomarkers in body fluids.

Patients' age, gender, blood pressure, blood lipids, atrial fibrillation history, and stroke history were associated with the prognosis of AIS [8]. In recent years, more studies have found that some cells and molecules in body fluids play a key role in predicting the prognosis of AIS. Due to its less invasive and more accurate advantages, liquid biopsies have gained increasing interest from the medical and scientific communities over the past few years. The most frequently analyzed samples are peripheral blood, and recent evidence suggests that other body fluids, including urine, saliva and cerebrospinal fluid (Figure 1B), are of great importance in detecting and monitoring the prognosis of AIS.

Prior to this, people's interest in body fluid biomarkers for AIS mainly focused on diagnosis [9,10] and disease progression [11,12], but there was a lack of summary of prognostic-related body fluid biomarkers. It is worth noting that significant progress has been made in recent years in predicting neurological outcome, mortality, and post-stroke depression based on early body fluid biomarkers of AIS. In this study, we conducted a comprehensive search of published research articles in PubMed, Google Scholar and other databases using key phrases in the past decade, such as “acute ischemic stroke”, “prognosis”, “biomarker”, “body fluid” *et al.* We selected body fluids biomarkers with important prognostic value and summarized and discussed them. In the review, we reviewed the relevant literature on AIS prognostic biomarkers in body fluids, and discussed the value of detectable AIS prognostic biomarkers in different body fluids, and how they can be applied in clinical practice, such as prognosis assessment, recurrence detection of the early and minor diseases, and early intervention etc. Some typical biomarkers have found in body fluids (Figure 1C), including neutrophil–lymphocyte ratio (NLR) and C-reactive protein (CRP) in blood, baseline albuminuria (urinary albumin–creatinine ratio, UACR) and estimated glomerular filtration rate (eGFR) in urine, oral salivary microbial group in saliva, and neurogranular protein (Ng) in cerebrospinal fluid (CSF). These biomarkers have shown promise in predicting outcomes and guiding treatment decisions in AIS patients.

## Effect of blood biomarkers on prognosis of AIS

### Inflammatory marker

Inflammation is closely related to severity [13], neurological deterioration [14] and stroke recurrence of AIS [15]. It has been found that inflammatory substances released by active immune cells can destroy the blood–brain barrier and then infiltrate into ischemic brain tissue is the key pathological process of AIS [16]. These immune-inflammatory components play a critical role in AIS. Therefore, immune cells and inflammatory molecules in the blood can be used as prognostic markers of AIS.

#### Changes of immunocytes

Routine blood tests can effectively reflect the quantitative variations of various blood cell types, encompassing immune cells, and they are characterized by their simplicity and cost-effectiveness. Consequently, whole-blood immune cell counts or ratios have emerged as the most extensively biomarkers for AIS. Numerous studies have shown that immunocyte-related parameters can effectively predict the prognosis of AIS (Table 1). Neutrophils-to-lymphocytes ratio (NLR) has been extensively studied. Neutrophils first accumulate in peripheral blood after AIS occurs and destroy the blood–brain barrier by producing reactive oxygen species, proteases, and neutrophil extracellular traps (NETs) [16]. Therefore, neutrophils may be an important indicator of AIS disease progression and prognosis. Multiple studies have shown that a higher NLR at admission in patients with AIS was associated with a worse prognosis within 3 months of treatment [17,18]. For example, Higher admission NLR was significantly associated with mortality and safety outcomes in AIS patients receiving therapy [19,20]. Meanwhile, Sadeghi *et al.* have found that a high NLR-low LMR (lymphocyte-monocyte ratio) combination as observed at 24 h after thrombolysis can serve as an independent predictor of 3-months poor outcome in AIS patients [21]. Studies have also shown that high baseline NLR is an independent risk factor for poor prognosis in early neurodegenerative AIS [^22-24^]. To date, NLR is a reliable prognostic marker for AIS, and its prognostic value has been fully proven.

**Table 1.** Prognostic biomarkers of AIS associated with inflammatory and their prognostic value.

Biomarker	Detection time	Enrolled patients	Prognostic indicator	Ref.
NLR	On admission	First-ever AIS (n = 448)	Unfavorable functional outcome at 3 months (adjusted HR = 2.32)	[18]
Low NLR	After thrombectomy	AIS (n = 142)	Favorable outcomes at 3 months (OR = 0.785)	[20]
NLR	On admission	AIS received EVT (n = 211)	Early neurological deterioration (OR = 1.011)	[25]
PLR	On admission	AIS (n = 363)	Post-stroke depression (OR = 5.145)	[26]
High PLR and high NLR	On admission	First-ever AIS (n = 448)	Unfavorable functional outcome (adjusted HR = 3.77)	[18]
PNR	On admission	AIS (n = 151)	Unfavorable functional outcome (OR = 0.967)	[27]
PNR	24 h after IVT	AIS (n = 151)	Unfavorable functional outcome (OR = 0.933)	[27]
LMR	Before thrombolysis	AIS (n = 591)	Post-thrombolysis early neurological deterioration (OR = 0.680)	[24]
LMR	On admission	AIS (n = 179)	Unfavorable clinical outcome at 3 months (OR = 0.761)	[28]
Low LMR	On admission	AIS (n = 507)	Post-stroke depression (OR = 2.633)	[29]
WMR	On admission	AIS undergone vascular reperfusion surgery (n = 549)	Poor outcome at 3 months (OR = 2.257)	[30]
SII	On admission	AIS (n = 234)	Poor prognosis at discharge (adjusted OR = 2.350)	[31]
SII	On admission	Large vessel occlusion AIS received EVT (n = 379)	Symptomatic intracranial hemorrhage after EVT treatment (OR = 1.005)	[32]
SII	On admission	AIS received IVT (n = 216)	Poor functional outcome at 3-months (OR = 3.953)	[33]
Log-transformed SII	Admitted to ICU	AIS (n = 1181)	30–day all-cause mortality (HR = 2.44)	[34]
SIRI	On admission	AIS received EVT (n = 279)	Poor functional outcome at 3-months (OR = 1.08)	[35]
Low SIRI	On admission	AIS (n = 161)	Favorable outcomes at 3 months (OR = 1.557)	[36]
SIRI	On admission	Mild AIS (n = 240)	Unfavorable functional outcome at 3 months (OR = 2.938)	[37]
SIRI	On admission	AIS (n = 2043)	30–day all-cause mortality (HR = 1.54) 90-day all-cause mortality (HR = 1.56) 1-year all-cause mortality (HR = 1.44)	[38]
WBC	After IVT	AIS (n = 447)	Unfavorable functional outcome at 3 months (adjusted OR = 4.48) 90-day all-cause mortality (adjusted HR = 2.19)	[39]
Low WBC	On admission	AIS received IVT (n = 657)	3–month functional independence (OR = 1.69)	[40]
Leukocytosis 24 h	After IVT	AIS received IVT (n = 213)	Poor short-term functional outcomes (OR = 6.648)	[41]
High PCT	Within 48 h of admission	AIS (n = 748)	30–day all-cause mortality (HR = 5.486)	[42]
IL-5	On admission	First-ever AIS (n = 180)	Poor functional outcome at 3-months (OR = 0.042)	[43]
IL-6	On admission	First-ever AIS (n = 180)	Poor functional outcome at 3-months (OR = 1.293)	[43]
IL-6	After EVT	AIS received EVT (n = 210)	Early neurological deterioration (OR = 1.98)	[44]
IL-10	After EVT	AIS received EVT (n = 210)	Early neurological deterioration (OR = 1.18)	[44]
IL-10	On admission	AIS (n = 350)	Post-stroke depression (adjusted OR = 0.615)	[45]
Low IL-33	On admission	Non-thrombolytic AIS (n = 151)	Hemorrhage transformation (OR = 5.773)	[46]

CRP: C-reactive protein; EVT: Endovascular therapy; HR: Hazard ratio; IVT: Intravenous thrombolysis; LMR: Lymphocyte-to-monocyte ratio; NER: Neutrophil-to-eosinophil ratio; NLR: Neutrophil-lymphocyte ratio; OR: Odds ratio; PLR: Platelet-to-lymphocyte ratio; PNR: Platelet-to-neutrophil ratio; PCT: Procalcitonin; SII: Systemic immune-inflammation index; SIRI: Systemic inflammatory response index; WMR: White blood cell count to mean platelet volume ratio; WBC: White blood cell.

In addition to NLR, studies have found that platelet-to-lymphocyte ratio (PLR) played an important role in the development of AIS. Platelet aggregation usually increased the formation of AIS-related thrombosis and inflammation [16], thus studies have focused on the prognostic function of PLR. Such as Chen *et al.*, showed that baseline PLR is an independent prognostic factor for AIS. Moreover, a higher PLR indicates an increased risk of a poor prognosis of 3 months. However, compared with NLR, PLR exhibits a limited capacity to identify patients at high risk [18]. Conversely, a lower platelet-to-neutrophil ratio (PNR) at baseline corresponds to a more favorable prognosis for AIS [27,47]. Consequently, it can be inferred that neutrophil aggregation exerts a more substantial influence on the development of AIS.

White blood cell count (WBC) and white blood cell count-to-mean platelet volume ratio (WMR) are independent prognostic factors for poor outcomes in AIS [30,39,48]. White blood cells include lymphocytes, neutrophils, monocytes, eosinophils and other immune cells. However, lymphocyte-to-monocyte ratio (LMR) was an independent protective factor for AIS. Low LMR was significantly associated with poor prognosis for AIS [21,28,49], early deterioration of neurological function [24] and post-stroke depression [29]. This suggests that leukocytes are functionally diverse in AIS. In addition, studies have found that the reduction of eosinophils (AEC) is associated with the severity of AIS, poor prognosis, haemorrhagic transformation [^50-52^]. Relevant data indicate that higher neutrophils-to-eosinophils ratio (NER) predicts worse outcomes [53,54], but the protective mechanism of eosinophils remains unclear in AIS.

In addition, the immune inflammatory index (SII, SII = neutrophils × platelets/lymphocytes) can also predict the prognosis of AIS. For example, Huang *et al.*, showed that SII ≥1008.3 × 10^9^/l predicted a higher risk of death at discharge for AIS patients (OR = 2.350 95% CI: 1.149–4.803) [31]. Meanwhile, SII at admission was significantly associated with intracranial hemorrhage and deterioration of functional outcome after AIS recanalization [32,33]. Wu *et al.* found that Log-transformed SII was an independent modulator of 30-day all-cause death for AIS patients (HR = 2.44 95% CI: 1.72–3.46) [34]. The systemic inflammatory response index (SIRI, SIRI = neutrophils × monocytes/lymphocytes) was also an important prognostic factor for functional outcome in AIS. Chen *et al.* found that SIRI ≤2.54 × 10^9^/l at admission was an independent predictor of good prognosis after intravenous thrombolytic therapy [36], while other studies confirmed that SIRI at admission was significantly associated with 3-month all-cause mortality [38]. These findings suggested that SIRI was also an effective prognostic marker. In conclusion, the change of peripheral blood immune cells can effectively predict the prognosis of ALS.

#### Other inflammatory biomarkers

CRP and procalcitonin (PCT) are important indicators of inflammation in the body and are also commonly used items in blood biochemical tests. Increasing evidence suggests that blood concentrations of CRP and PCT are associated with AIS prognosis, and usually higher CRP or PCT in plasma are associated with AIS prognosis [55,56]. Interleukin is an important mediator for regulating inflammatory response, with complex functions in AIS prognosis. Studies have found that IL-5 and IL-6 are all independent prognostic factors for AIS. Recent studies have consistently shown that poorer patient prognosis is associated with higher levels of these cytokines (Table 1). IL-33 was an independent prognostic protective factor for AIS. Low IL-33 indicated a poorer outcome and a higher risk of post-stroke depression or bleeding [46,57,58]. The prognostic value of IL-10 in AIS is controversial. Low level of IL-10 in plasma were associated with poor prognosis in AIS and increased risk of post-stroke depression [45,59], but high levels of IL-10 are also risk factors for early neurological deterioration in AIS [44]. The function of IL-10 in AIS needs to be further explored. NETs can activate platelets to promote thrombosis. Elevated levels of NET marker citrullinated histone H3 (CitH3) in plasma have been reported to be associated with increased atrial fibrillation and all-cause mortality within one year of AIS [60]. These studies initially illustrate the prognostic value of inflammatory markers in the blood in AIS.

### Nucleic acid biomarkers

Earlier studies have shown that plasma DNA concentration can be used to predict mortality in AIS [61]. It suggests that blood nucleic acids may be prognostic markers for AIS. With the development of next-generation sequencing technology, scientists have found that a variety of non-coding RNAs are involved in the development of AIS and may be used as targets for AIS therapy [62]. Several microRNAs and lncRNAs have been used as markers for assessing the prognosis of AIS. Such plasma *miR-124-3p*, *miR-125b-5p* and *miR-192-5p* levels at 24 h after thrombolytic therapy were significantly related to the prognosis of AIS patients. It can be used as an independent prognostic factor for the poor prognosis of AIS [63]. In addition, *miR-9-5p*, *miR-128-3p*, *miR-491-5p* can also predict the prognosis of AIS (Table 2). High levels of *LncRNA ZFAS1* were a prognostic protective factor in patients with AIS [64]. *LncRNA NEAT1*, *LncRNA UCA1* and *LncRNA GAS5* were used as risk factors to predict RFS in patients with AIS (Table 2).

**Table 2.** Nucleic acid-related prognostic markers of AIS in blood.

Biomarker	Detection time	Enrolled patients	Prognostic indicator	Ref.
*miR-124-3p, miR-125b-5p, miR-192-5p*	24 h after thrombolysis	AIS (n = 84)	Unfavorable outcomes at 3 months	[63]
*miR-9-5p* and *miR-128-3p*	/	AIS (n = 88)	*miR-9-5p* and *miR-128-3p* were positively correlated with mRS score (r values were 0.066 and 0.082, respectively)	[65]
*miR-491-5p*	On admission	AIS (n = 215)	Unfavorable outcomes at 1 year (OR = 0.9)	[66]
*miR-206*	On admission	AIS (n = 215)	Hemorrhagic transformation (OR = 1.64)	[66]
*miRNA-24* and *miRNA-29b*	/	AIS (n = 105)	*miR-24* and *miR-29b* were negatively correlated with NIHSS score (r values were -0.758 and -0.794, respectively) and mRS score (r values were -0.817 and -0.860, respectively) in elderly AIS patients	[67]
*miR-185* and *miR-424*	On admission	AIS (n = 142)	*miR-185* and *miR-424* were positively correlated with NIHSS score and mRS score in AIS patients (r values were0.735, 0.802, 0.796, 0.873)	[68]
*LncRNA NEAT1*	On admission	AIS (n = 210)	RFS in *lncRNA NEAT1* high expression patients was shorter compared with *lncRNA NEAT1* low expression patients	[69]
*LncRNA UCA1*	On admission	first-episode AIS (n = 160)	High *lncRNA UCA1* expression was associated with worse accumulating RFS in AIS patients	[70]
*LncRNA GAS5*	On admission	AIS (n = 120)	Elevated *lnc-GAS5* was correlated with shorter RFS	[71]
*LncRNA HULC*	On admission	first-episode AIS (n = 215)	*lncRNA HULC* high expression predicted worse RFS	[72]
*LncRNA NORAD*	On admission	AIS (n = 103)	*NORAD* upregulation was associated with unfavorable prognosis; *NORAD* was positively correlated with NIHSS scores (r = 0.840)	[73]
*LncRNA ZFAS1*	On admission	AIS (n = 241)	Lower *lnc-ZFAS1* expression relates to increased neurological impairment and inflammation as well as worse RFS in AIS patients	[64]
*MALT1*	On admission	first-episode AIS (n = 120)	High *MALT1* expression independently associated with shorter RFS in AIS patients	[74]
cfDNA	On admission	AIS (n = 54)	Higher cf-DNA levels were associated with poor outcome in AIS patients	[75]

cfDNA: Cell-free DNA; HR: Hazard ratio; *MALT1*: Mucosa-associated lymphoid tissue lymphoma translocation protein 1; OR: Odds ratio; r: Person correlation coefficient.

In addition, cell-free DNA (cfDNA) in the blood was also a prognostic factor for AIS. There was cellular damage and blood–brain barrier disruption in AIS patient, which resulted in the release of cfDNA into the blood. Studies have shown that higher cfDNA levels in plasma of patients with AIS lead to worse outcome [75]. Blood-specific lncRNA or microRNA was more stable and less demanding than cfDNA, and they may be a more promising prognostic marker.

The imperative of swiftly handling and analysing blood samples arises from the inherent instability of miRNA, lncRNA, mRNA and ctDNA. Simultaneously, these biomarkers harbour valuable genetic expression and regulatory information, enabling precise real-time cell status assessment. With the support of high-throughput sequencing technology and real-time fluorescence quantitative PCR, it will surely become an important biomarker for prognostic management of AIS.

### Other biomarkers in the blood

In addition, plasma endostatin [76], brain natriuretic peptide (BNP) [77,78], serum ferritin [79,80], plasma fibroblast growth factor [81], plasma trimethylamine-N-oxide content [82], hypersensitive cardiac troponin [83], coagulant-related factors C-type lectin receptor 2 and anti-phosphatidylserine [84,85], circulating IGF-1 [86], plasma lipid metabolism [^87-89^], plasma D-dimer [90] and other biochemical metabolites may be related to the prognosis of AIS. For example, Yang *et al.* prospectively included patients with AIS pneumonia associated with atrial fibrillation (AF) and found significant increases in 3-month mortality and severe residual risk associated with high D-dimer levels and pneumonia AIS, suggesting that plasma D-dimer has prognostic value [91]. Wu *et al.* found that plasma neurofilament light chain (pNfL) levels in ischemic stroke patients were significantly elevated at 2 days, 7 days and 6 months after stroke compared with healthy controls, and pNfL levels varied over time, suggesting that pNfL may be a promising biomarker for predicting severity in patients with acute neuroaxonal injury of AIS [92]. In addition, researchers found that serum neuron-specific enolase concentrations on the second day of admission predicted long-term functional outcomes at 1 year in patients with hypertensive AIS [93].

Recently published studies presented the crucial role of brain-derived neurotrophic factor (BDNF) in ischemia. BDNF is a member of the neurotrophins family that has a crucial role in the development and maintenance of the nervous system. BDNF regulates neurotransmission, neuronal regeneration and morphology, and functional synaptic plasticity in peripheral and central nervous system (CNS) neurons through binding to tropomyosin-related kinase B (TrkB) [94,95]. It was observed that BDNF levels during the first 24 h of stroke were significantly higher among patients under 65 years compared with older individuals. Additionally, low BDNF concentrations were associated with clinical status during 90-day follow-up [96]. It indicated that BDNF levels in the acute phase of ischemic stroke may possess a prognostic value [97,98]. Excitingly, some studies are beginning to increase BDNF expression to promote recovery from ischemic stroke [99]. Recent studies have found that protein glycosylation, particularly O-GlcNAcylation played a crucial protect role in the occurrence and outcome of AIS [100]. Drugs target enhanced protein glycosylation such as glucosamine and thiamet-G have demonstrated beneficial effects on ischemic stroke outcomes in stroke models [100]. These studies suggest that conversion of prognostic markers into therapeutic targets can provide better disease management strategies for AIS.

## Prognostic markers of AIS in other body fluids

### Urine

Besides the blood related markers, these markers in urine were also associated with the prognosis of AIS. Compared with blood samples, urine is easier to obtain and process.

#### Protein markers in urine

High albuminuria has been significantly associated with an increased risk of stroke and unfavorable long-term outcomes. For example, high UACR on admission may predict poor outcome among patients with AIS [101,102]. Strain *et al.* used albuminuria as an outpatient prognostic marker for transient ischemic attack (TIA) or mild stroke [103]. Wang *et al.* have screened a total of 14015 patients diagnosed with either AIS or TIA, showing that urinary ketone positivity predicted all-cause death and adverse functional outcomes in an independent manner [104]. Furthermore, Cao *et al.* highlighted the importance of urinary protein and urinary ketone as indicators for short-term prognosis of AIS patients [105]. Besides, Nakanishi *et al.* revealed that Urinary titin rapidly increased after stroke and was associated with impaired functional outcomes at hospital discharge (Table 3) [106]. Therefore, the urine dipstick testing (UDT) has been used as a preliminary screening tool of proteinuria to predict clinical outcomes [107]. In the future, the development of urine protein detection products for prognostic management of AIS may be an important means to simplify prognostic management.

**Table 3.** Prognostic biomarkers in urine, saliva, and CSF.

Biomarker		Detection time	Enrolled patients	Relative prognostic value	Ref.
Urine	Urinary protein	On admission	AIS (n = 2842)	Patients with positive urinary protein was associated with a 2.74-fold and 1.62-fold increase in the risk of in-hospital mortality (adjusted HR = 2.74)	[105]
	UACR	On admission	AIS (n = 294)	Early neurological deterioration was positively associated with high UACR (≥39.6 mg/g creatinine)	[101]
	Urinary ketone	On admission	AIS or transient ischemic attack (n = 14015)	30–day all-cause mortality (HR = 2.44), Poor functional outcomes (OR = 1.85	[104]
	Urinary titin	Immediately after admission, and on days 3, 5, and 7	AIS (n = 41)	The peak urinary titin level was associated with the mRS score (r = 0.55), the NIHSS score (r = 0.72), and the Barthel index (r = -0.59) upon hospital discharge	[106]
Saliva	g_Streptococcus, g_Prevotella, g_Veillonella, and g_Fusobacterium	2 h after ischemic stroke	AIS (n = 146)	The higher the abundance of two specific microorganisms, the worse the prognosis of AIS patients at 3 months	[108]
CSF	Tau	On admission	AIS (n = 50)	CSF tau was also significantly correlated with mRS score at 12 months after stroke (r = 0.403)	[109]
	GFAP, S100B	On admission	AIS (n = 89)	GFAP and S100B concentrations correlated with mRS score at month 3 (r = 0.42 and r = 0.28, respectively).	[110]
	CSF/plasma proCPU ratio	Hyperacute phase	AIS (n = 72) or transient ischemic attack (n = 14)	statistically significant correlations of the proCPU ratio with mRS score and mortality at month 3 (r = 0.30 and r = 0.31, respectively)	[111]

CSF: Cerebrospinal fluid; GFAP: Glial fibrillary astrocytic protein; HR: Hazard ratio; OR: Odds ratio; proCPU: Procarboxypeptidase U; r: Person correlation coefficient; Tau: Microtubule-associated protein tau; UACR: Urinary albumin/creatinine ratio.

#### Other markers in urine

Furthermore, inadequate hydration is believed to increase the risk of vascular disease, and blood and urine parameters have been used to determine a patient's hydration status. Stella *et al.* tested the urinary osmotic pressure (uOsm) in stroke patients, indicating that uOsm may be a factor associated with stroke severity and independence after acute ischemic stroke [112,113]. Ling *et al.* found that exosome from human urine-derived stem cells to enhanced neurogenesis via *miR-26a*/*HDAC6* axis after ischaemic stroke [114]. Taken together, this study indicates that exosome can be used as a novel promising strategy for brain ischaemia.

### Saliva

In recent years, several studies have demonstrated that alteration in gut microbiota composition influences the outcomes of ischemic stroke. Interestingly, many of the oral pathogenic bacteria in the saliva that are swallowed and transmitted to the gut can affect ischemic stroke [115]. Wu *et al.* recruited observational studies on three groups of subjects (IS, high-risk IS [HRIS] and healthy control [HC]) and found that HRIS and the oral salivary microbial group of IS subjects had a high diversity and predictive value in the severity and prognosis of IS [108]. Therefore, oral microflora may serve as a potential biomarker for patients with IS. Mechanistically, periodontitis salivary flora can affect the intestinal immune system, leading to accumulation of Th17 cells and IL-17+γδT cells aggregating in the gut and exacerbating ischemic stroke. furthermore, the worse stroke outcome was abolished in the IL-17A knockout mice, suggesting that increased IL-17A is associated with exacerbation of AIS [115].

Inflammation plays a crucial role in stroke pathogenesis. higher saliva matrix metalloproteinase-8, myeloperoxidase, IL-1β, *Aggregatibacter actinomycetemcomitans* in saliva of AIS patients [116]. Recent studies have revealed that individuals with the TT genotype of the *MMP-9*-1562C/T polymorphism may have an increased susceptibility to hemorrhagic complications associated with rtPA-induced AIS [117]. Maciejczyk *et al.* showed that salivary TNF-α and IL-6 were significantly higher in whole saliva of ischemic stroke patients. Of particular note is salivary TNF-α, which may differentiate stroke patients with normal cognition from mild to moderate cognitive impairment [118]. Furthermore, stroke is one of the most common causes of cognitive impairment in people over 65 years of age, and hyperactivation of xanthine oxidase (XO) activity is negatively related to cognitive performance in stroke patients. XO-specific activity in whole saliva can distinguish stroke patients with mild to moderate cognitive decline with high accuracy (100%) and specificity (93.75%) [119].

In previous studies, it was found that some specific markers in the brain were more closely related to saliva than to blood [^120-122^]. These results suggest that saliva samples are attractive detection samples for AIS. However, there are still few saliva-related prognostic markers for AIS, and large-scale clinical trials are needed to further prove it.

### Cerebrospinal fluid

#### Protein markers in cerebrospinal fluid

Ng is a small protein usually expressed in granule-like structures in pyramidal cells of the hippocampus and cortex. Ng is a potential and promising biomarker to improve the prognosis of AIS [123]. Vincent *et al.* conducted a systematic review and found that changes in protein concentrations associated with cytoskeletal injury, inflammation, apoptosis and oxidative stress were linked to poorer neurological outcomes in patients with acute brain injury [124]. In addition, some studies have proven that markers such as microtubule-associated protein tau (Tau) and GFAP in cerebrospinal fluid are also significantly related to the functional outcomes of AIS patients (Table 3).

#### Cytokine marker in cerebrospinal fluid

Post-stroke cognitive impairment (PSCI) is a clinically heterogeneous disease. Kulesh *et al.* measured and analyzed the concentrations of cytokines (IL-1β, IL-6, TNF-α, IL-10) in cerebrospinal fluid and serum from 92 patients, as well as some MRI morphological parameters and fractional anisotropy. They found that concentrations of these cytokines may be biomarkers of clinical types of post-stroke cognitive impairment [125].

Compared with blood, urine and saliva, obtaining cerebrospinal fluid is more invasive. More research in the future can focus on the consistency of cerebrospinal fluid test results with other body fluids to overcome the difficulties of cerebrospinal fluid sampling.

## Conclusion

AIS is a diverse disease with high morbidity, disability, mortality and recurrence rates. Therefore, the identification and development of prognostic biomarkers to accurately assess disease progression in patients with AIS is of great importance to improve the quality of life. Liquid biopsy is considered as a promising method of molecular diagnosis due to its accuracy and non-invasiveness. A large number of available AIS humoral biomarkers that play an important role in predicting and improving the prognosis of AIS. Some of these immune cell-related prognostic markers have already been used in AIS clinical practice, and immune cell-based prognostic assessment has a broad application foreground. The liquid biopsies for AIS detection, diagnosis and disease monitoring are exciting prospect.

## Future perspective

Since AIS patients need secondary prevention immediately after stroke, it is particularly important to determine their risk of recurrence for preventive management. However, there are still many practical issues that need to be addressed. First, although a number of studies have reported many prognostic influences on AIS in recent years, especially biomarkers in body fluids, most of the prognostic factors lack sufficient evidence and need to be proven in further clinical trials. Secondly, the prognostic mechanisms of those mentioned biomarkers for AIS are still inadequate. In addition, the medical conditions and treatment techniques for AIS treatment varied among regions, which may also lead to significant differences in the degree of influence of their prognostic factors when studied.

Furthermore, the prognostic performance of each biomarker is different. Comparison of each biomarker can screen out excellent biomarkers. A comprehensive assessment of multiple parameters enables a holistic evaluation of the patient's condition and prognosis from diverse perspectives, thereby enhancing the precision and sensitivity of predictive models. Consequently, clinicians acquire a more comprehensive insight into the patient's data, facilitating the formulation of customized treatment strategies. The implementation of this detection methodology improves the efficacy of assessing the patient's condition and prognosis, ultimately leading to the development of comprehensive treatment strategies.

The outcome of stroke patients can be significantly improved by thoroughly studying the prognostic biomarkers associated with AIS and developing reasonable prognostic models, which in turn will provide more possibilities for individualized interventions for improvement. As the research on biomarkers in body fluids in predicting the prognosis of AIS continues, it is believed that their application in clinical practice of AIS prognosis will become more and more widespread in the future.
